# Cardiac amyloid burden assessment by T1 mapping predicts survival in patients with systemic AL amyloidosis - a 2 year follow-up study

**DOI:** 10.1186/1532-429X-16-S1-O5

**Published:** 2014-01-16

**Authors:** Sanjay M Banypersad, Marianna Fontana, Viviana Maestrini, Daniel Sado, Steven K White, Thomas A Treibel, Helen Lachmann, Ashutosh Wechalekar, Philip N Hawkins, James Moon

**Affiliations:** 1 MRI, The Heart Hospital, London, UK; 2 NAC, Royal Free Hospital, London, UK

## Background

Cardiac involvement drives outcome in systemic AL amyloidosis. Late gadolinium enhancement (LGE) CMR is useful for the detection of cardiac amyloid, but characteristic LGE patterns do not always occur or may appear late in the disease. Using CMR T1 mapping, we measured the pre contrast myocardial T1 and myocardial Extracellular Volume (ECV), reflecting myocardial amyloidosis burden and determined their prognostic significance.

## Methods

102 patients underwent CMR and T1 mapping pre- and post contrast. Myocardial ECV was calculated at contrast equilibrium. 54 healthy volunteers served as controls. Patients were followed up for a median duration of 23 months and survival analyses were performed. A secondary analysis compared predictive power of the following techniques: pre contrast T1, post contrast T1, ECV at equilibrium (ECVi) and ECV at 15 minutes i.e. bolus only ECV (ECVb).

## Results

ECVi and ECVb were both raised compared to normals (both ECVi and ECVb means: 0.44 ± 0.12 vs 0.25 ± 0.02 for healthy volunteers, P < 0.001), as was native T1 (1081 ms vs 954 ms, P < 0.001). All 3 tracked pre-test probability of cardiac involvement, cardiac biomarkers and systolic and diastolic dysfunction. During follow-up, 25 deaths occurred. An ECVi of > 0.44 carried a hazard ratio for death of 3.76 (95% CI: 1.50 - 9.43), P = 0.005 and pre contrast T1 of > 1080 ms = HR 3.38 (95% CI: 1.21 - 9.45), p = 0.020. ECVi and ECVb performed similarly. Both ECVi and ECVb were stronger predictors of survival than pre contrast T1 as assessed by the (Harrell's C statistic). Isolated post contrast T1 was non predictive.

## Conclusions

Myocardial ECV (bolus or infusion technique) and pre contrast T1 in AL amyloidosis are strong predictors of mortality with ECV (however measured) being the better predictor.

## Funding

GSK.

**Figure 1:**
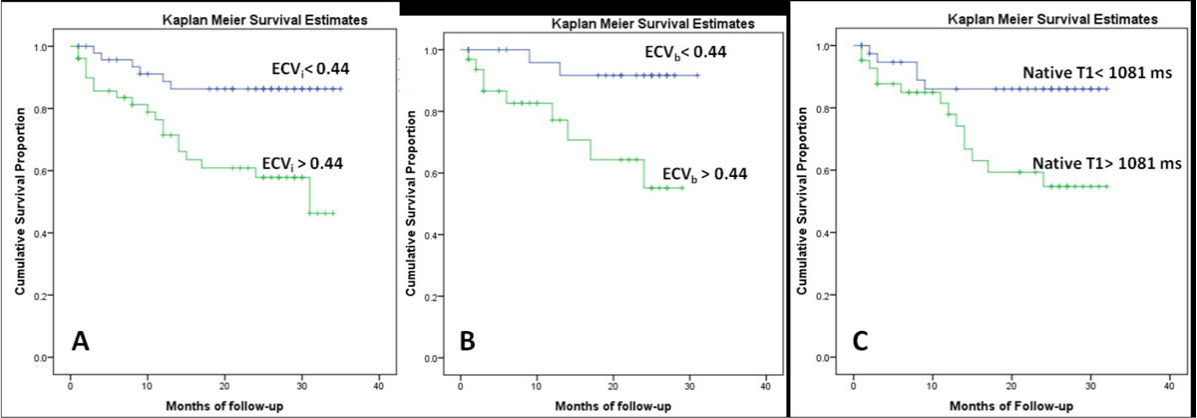
**Kaplan Meier survival curves after median duration of follow up of 23 months for: (A) ECVi; (B) ECVb; (C) pre-contrast myocardial T1**.

